# Prevalence of Intimidation of Medical Students in the Clinical Years by the Staff at a Saudi Arabian University

**DOI:** 10.7759/cureus.51508

**Published:** 2024-01-02

**Authors:** Bandar M Hetaimish

**Affiliations:** 1 Orthopaedic Surgery, College of Medicine, University of Jeddah, Jeddah, SAU

**Keywords:** saudi arabia, learning, abuse, medical students, intimidation

## Abstract

Background: Intimidation of medical students by the staff appears to be an evolving problem. Our study aimed to determine the prevalence of intimidation among medical students by the staff, identify the types of intimidation that exist, and recognize the barriers that stand in the way of reporting any incident of intimidation.

Methods: A cross-sectional study was conducted among medical students in their clinical and final years at the College of Medicine, Taibah University (TU), Al-Madinah Al-Munawwarah, Saudi Arabia. Students were invited to voluntarily complete an anonymous survey that explored students’ exposure to any type of intimidation, including verbal, physical, and sexual abuse, and academic intimidation.

Results: A total of 311 medical students participated in our study, with 164 females and 147 males. Of these, 187 (60.1%) students reported having received some sort of intimidation. The most testified type of intimidation was verbal abuse (165, 53.1%). Following that, in order of frequency, were academic intimidation (101, 32.5%), physical intimidation (25, 8.0%), and sexual abuse (13, 4.2%).

Conclusion: Our study revealed a high rate of intimidation between the staff and medical students at our institution. Verbal abuse was the most popular type of intimidation. Promoting a more positive learning environment for medical students by managing the teacher-student relationship is crucial to improving students’ learning outcomes. Medical schools should resist intimidation through strict guidelines.

## Introduction

Intimidation is premeditated conduct directed towards a person that creates a fear of injury or harm to that person. It is not necessary to prove that the conduct was forceful to cause fear or that the victim was actually terrified [1]. In our study, “intimidation” is defined as “any action toward medical students that is frightening, threatening, or forces them to do something resulting in physical, sexual, emotional, or financial harm.”

Common reported forms of intimidation of medical students include verbal abuse or humiliation, nonsexual and sexual harassment, exclusion from or denial of access to opportunities, and/or undue additions to work requirements [2]. In addition, the power that a mentor has over a mentee could create an environment for abuse. Several forms of abuse have been reported in various occupational settings, including the healthcare sector [3, 4].

In general, the prevalence of intimidation in medical schools has wide variability that might be related to the region or culture. Alzahrani investigated bullying among 542 clinical-year medical students at the King Abdulaziz Medical School in Jeddah, Saudi Arabia, through a cross-sectional study. It was found that more than 28.0% of the surveyed students declared exposure to some form of bullying during their clinical years. A total of 90% of the declared insults were verbal, 6% were sexual, and 4% were physical. The percentage increased significantly with the growth of students from the fifth year (18.5%) until it reached its peak in the internship year (43.7%). Males were more exposed than females. However, the percentage of females who had been exposed to sexual harassment was higher than that of males (9.8% vs. 3.4%) [5].

Students’ intimidation affects the medical school environment negatively. The barriers that prevent the students from reporting any intimidation are not clear, masking the real prevalence of intimidation. Some of these barriers are associated with cultural variance, academic grades, official responsibility, or even other causes [6]. This study aimed to determine the prevalence of intimidation among medical students at Taibah University (TU), Saudi Arabia, identify the types of intimidation, and assess the barriers to reporting any incident of intimidation.

## Materials and methods

Study design and participants

We conducted a cross-sectional study among medical students in clinical and final years (third, fourth, and fifth) at the College of Medicine, Taiba University, Al-Madinah Al-Munawwarah, Saudi Arabia. All clinical-year students who had direct contact with physicians in hospitals were included. Medical students in their basic years were excluded. Participants were invited to voluntarily complete an anonymous survey.

Survey

An online questionnaire was developed, taking into consideration the survey that was previously done at King Abdulaziz University [5]. The survey was open for two weeks. A reminder email was sent after one week requesting completion of the survey. The survey consisted of 13 questions: 11 Likert scale close-ended questions, one multiple-answer question, and one open-ended question. The first two questions provided a general idea about the prevalence of intimidation. Questions three, four, and five concentrated on verbal abuse. Questions six and seven inquired about physical abuse. Questions eight and nine focused on academic abuse. Question 10 targeted sexual abuse. Question 11 delineated medical students’ responses to any abuse. Question 12 highlighted the barriers for a student when reporting the incident. Participants were given the opportunity to select more than one answer. The last question was an open-ended question where the respondent could provide further details on staff intimidation. The questionnaire is provided in Appendix A.

Ethical consideration

This study was approved by the College of Medicine Research Ethics Committee, TU (approval number: 080316). Informed consent was obtained from participants as this was a questionnaire-based study. Confidentiality of data was guaranteed by the use of codes for all study subjects included in this research.

Statistical analysis

The data were analyzed using IBM SPSS Statistics, version 23 (IBM Corp., Armonk, NY).

## Results

Out of 453 students at TU, a total of 311 (68.7%) medical students participated in our study, with an estimated 142 (31.3%) non-respondents. Gender distribution in the study was almost equal between males (147, 47.3%) and females (164, 52.7%). The number of medical students participating in the study according to their clinical years was as follows: third year, 92 (29.6%); fourth year, 113 (36.3%) and fifth year, 106 (34.1%).

Prevalence of abuse

Results showed that there was a significantly low incidence of receiving any type of intimidation at the university: sexual, 9.7%; physical, 15.1%; and academic, 46.3%, except for verbal intimidation, which had a high incidence (67.2%). In general, the majority of students (around 71%) would not report being exposed to any intimidation in their clinical years because of a variety of barriers, most ranging from fear of losing grades by the doctors (76.5%) to no response from the officials (50.3%).

**Figure 1:**
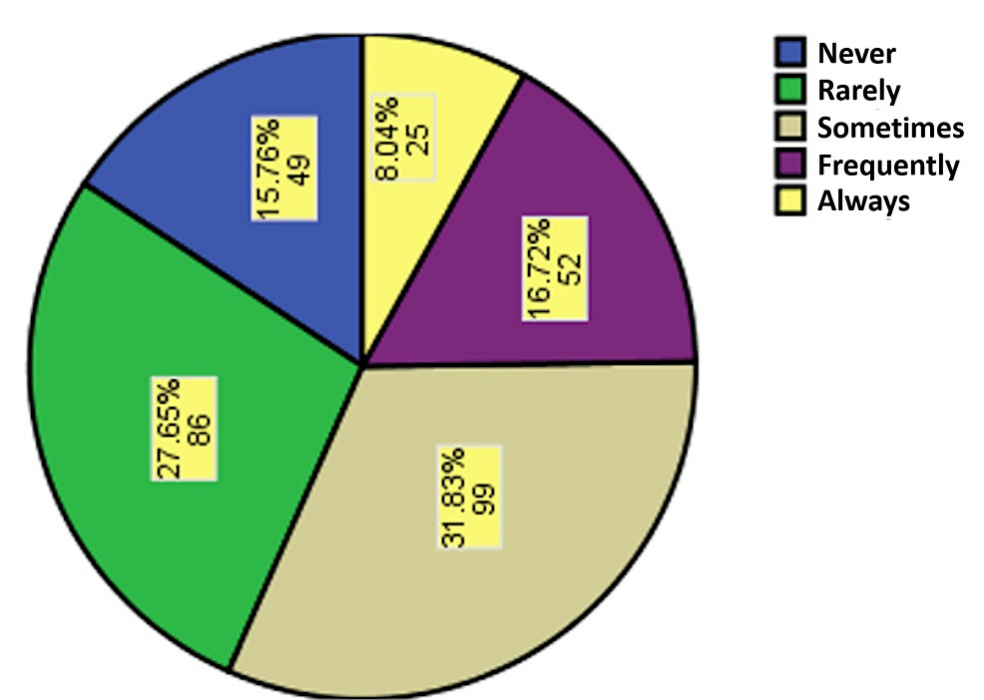
Percentage of respondents who had heard about the intimidation phenomenon in the medical student fraternity

There were 99 students (31.8%) who reported that they sometimes heard about intimidation from the staff, while 52 (15.8%) students reported that they had never heard about staff intimidation (Figure 1).

Verbal intimidation

**Figure 2:**
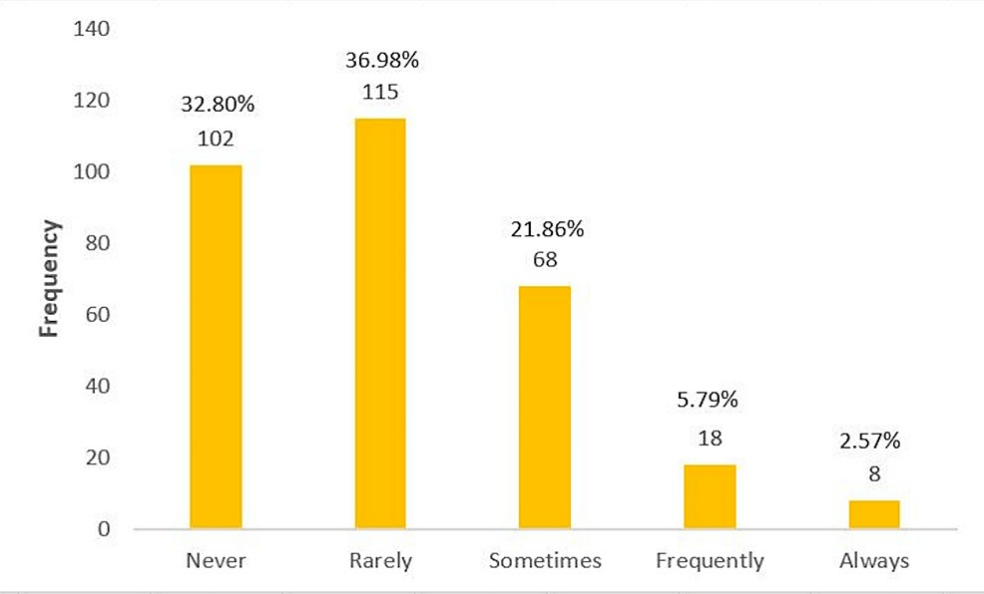
Frequency of verbal intimidation

Verbal intimidation refers to any kind of shouting or speaking in an unpreferable language. We had 115 (37.0%) participants who stated that they rarely encountered verbal intimidation in their clinical years. On the other hand, 26 (8.4%) participants mentioned that they received verbal intimidation in their clinical years, further divided into frequently (5.8%) and always (2.6%) (Figure 2).

Physical intimidation

Physical intimidation refers to any kind of physical harm or threat from the staff to medical students, even if it is from a distance and without contact. The majority of the participants (264, 84.9%) reported that they never faced any kind of physical intimidation from the staff. However, 47 (15.1%) students reported physical harassment from the staff at their medical school (Table 1).

**Table 1:** Frequency of physical intimidation

	Frequency	Percentage
Never	264	84.9
Rarely	22	7.1
Sometimes	19	6.1
Frequently	4	1.3
Always	2	0.6
Total	311	100.0

Academic intimidation

Academic intimidation is defined as any threat to the students of losing grades or even failing the class. The results in this study varied from never facing academic intimidation (53.7%) to a combined “frequently” and “always” receiving academic intimidation (5.2%).

Sexual intimidation

Any kind of unwanted touching, including patting, poking, stroking, hand touches, or even more than that, is considered sexual intimidation. Our study showed a low incidence of receiving any kind of sexual intimidation, as 90.0% of the students selected “never” as an answer when asked if they had received sexual intimidation in TU, and 1.3% of the medical students had encountered sexual intimidation from the staff.

Responding to intimidation

**Figure 3:**
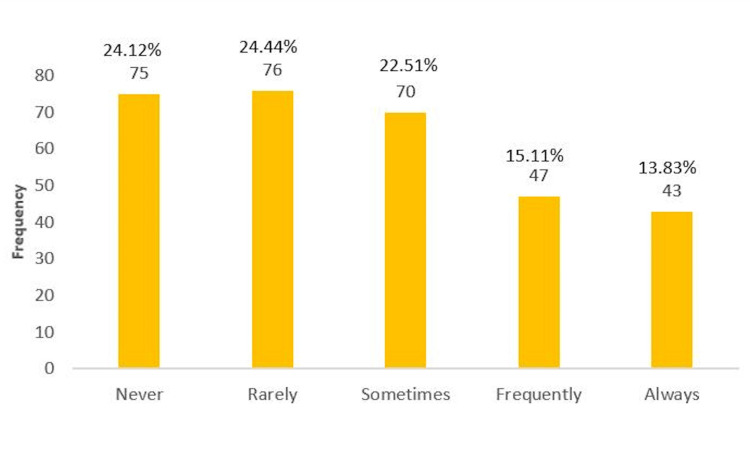
Frequency of participants reporting an intimidation incident

We also wanted to know how often the students would report an intimidation incident. The results of reporting the incident were scattered between never (24.1%), rarely (24.4%), sometimes (22.5%), frequently (15.1%), and always (13.8%). However, the majority of students (71%) leaned toward not reporting if they received intimidation (Figure 3).

Barriers preventing the students from reporting intimidation

**Figure 4:**
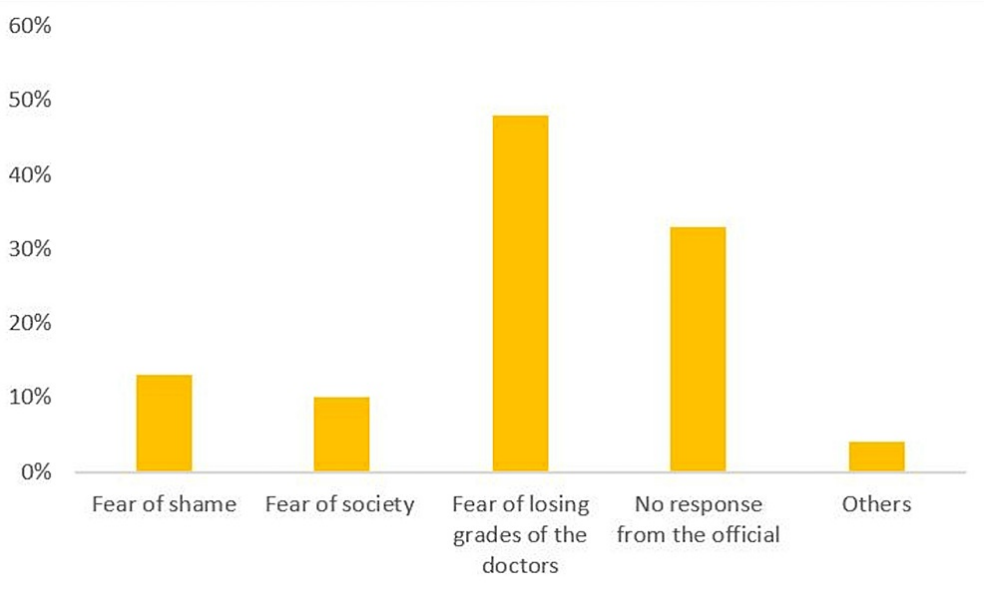
Different barriers that prevent medical students from reporting intimidation Data are presented as percentages.

As the majority (71%) would not report if they received any kind of intimidation, we searched for the barriers that may prevent the students from reporting. They were as follows: fear of losing grades by the doctors (44%), no response from the officials (31.48%), fear of shame (12.2%), fear of society (9.7%), and other barriers (2.62%) (Figure 4).

## Discussion

The intimidation of students by the staff appears to be a worldwide problem these days, especially in the healthcare sector, and there are not enough studies on this in many countries. Hence, we decided to apply this research to clinical year medical students at TU to uncover the prevalence of intimidation by staff, and types of abuse, and assess the barriers to reporting any intimidation.

We found that 44 (14.1%) out of 311 students experienced abuse at least once at TU during their clinical years. Comparatively, verbal (67.3%) and academic abuse (46.3%) formed the largest percentage of the mistreatment, followed by sexual (10%) and physical abuse (15.11%), which had lower percentages. A total of 71% of the respondents testified that they would not report the abuse if they experienced it, with the fear of losing grades by the doctors (44%) being the main reason. In comparison, in research from Jeddah, Saudi Arabia, that included 542 medical students, the prevalence of abuse was 28.0% [5]. A similar study was performed in Jordan, where it was found that 61% of the medical students had been exposed to at least one of the following types of harassment: verbal (52%), physical harm (32%), and sexual harassment (33%) [6]. A study was conducted among interns at King Abdulaziz University, Jeddah, that revealed that 90.9% of 169 candidates reported experiencing some form of abuse during medical school training. It was most often verbal (86.6%), followed by academic abuse (73.1%), sex discrimination (38.7%), racial or ethnic discrimination (29.0%), physical abuse (18.8%), religious discrimination (15.1%), and sexual harassment (8.6%) [2]. In another study that included final-year medical students from six medical colleges in Pakistan, 52% of respondents reported that they had been exposed to some sort of harassment during their medical education. The most frequent form of harassment was verbal abuse (57%), while consultants were the most frequent perpetrators (46%) [7].

Rautio and colleagues studied the mistreatment experienced by medical students by the staff and fellows at the University of Oulu, Finland. Their results showed that 40% of men and 55% of women had been exposed to some harassment by staff; 21% of students reported at least one type of harassment, and 12.6% reported four or more different types of harassment. The most common form was belittlement and humiliation (40%), followed by disparaging remarks about the respondent's academic performance (34%), yelling and shouting (23%), sexual harassment (17%), and tasks assigned as punishment (13%) [8]. Furthermore, Rademakers et al. investigated sexual harassment during clinical clerkships in Dutch medical schools. Their results showed that one in three to five Dutch female medical students had been exposed to sexual attention from patients, colleagues, or supervisors. However, these were low prevalence rates compared to international rates [9].

Another healthcare intimidation study was performed among residents in three training hospitals in Saudi Arabia. At least one form of harassment was reported by 83.6% of respondents, which were 213 out of 380 residents. The most common forms were verbal harassment and gender discrimination (61.5% and 58.3%, respectively). Sexual harassment was reported by 19.3% of respondents, and females experienced sexual mistreatment more often than male residents [10].

There are some similarities between our study and previous research. One is the highest percentage of mistreatment found for verbal intimidation between staff and medical students. Moreover, our results are aligned with the results of the research conducted among interns at King Abdulaziz University in Jeddah, where both studies found the lowest percentage response for sexual abuse [2].

Most students idolize their professors and want to follow not only their academic characteristics but also their behavioral characteristics. Teachers or hospital staff with sarcastic attitudes toward students may create negative attitudes in students toward their profession [11]. Furthermore, most of the students who carry negative attitudes and have suffered abuse may become abusers themselves. Kluft showed the possibility that medical student abuse may contribute to future patient abuse [12]. In addition, Silver and Glicken suggested the similarity between medical student abuse and child abuse [13].

As in other local and international studies, the prevalence of intimidation among medical students at TU appears to be high. This is a problem that emotionally affects the students, their performance, and their learning outcomes, and may affect their future careers as doctors. This matter requires further attention and additional studies to identify the actual causes and offer solutions for providing a safe and professional teaching environment.

There are some limitations to this cross-sectional study. First, even though medical student abuse was clearly defined in our questionnaire, the judgment on the incident may vary from one person to another. Second, we do not have mistreatment information about the non-respondents (31.3%), and it is possible that non-respondents' experiences may differ in ways that bias our findings. For example, if students with high rates of mistreatment were more likely to respond, our estimate of the prevalence of mistreatment may be too high, and vice versa. Third, the mistreatment is subject to biases such as recall bias that may lead to wrong results because the response was only reported by the students themselves and there was no other party. Fourth, we do not know the exact location of the mistreatment, whether it was in the hospital or at the university, or the academic level of the staff. This could affect the result, as different affiliated staff might be involved in the incident. Additionally, there are other psychological and mental health factors, such as stress, depression, and coping skills, that we did not measure in our study. This is sometimes reflected in students' overreporting of any abuse incident against their teachers.

## Conclusions

Our study revealed a high rate of intimidation between staff and medical students at our institution. Verbal abuse was the most popular type of intimidation. Promoting a more positive learning environment for medical students by managing the teacher-student relationship is crucial to improving students’ learning outcomes. Medical schools should resist intimidating behavior through strict guidelines.

## Appendices

Appendix A

This survey was done to study the incidence of intimidation between staff and medical students in clinical years at TU, Saudi Arabia, the types of intimidation, and the perceived barriers to reporting that intimidation.

Intimidation is defined as “any action toward medical students that is frightening, threatening, or forces them to do something resulting in physical, sexual, emotional, or financial harm.”

Questionnaire

**Table 2:** Questionnaire used for the study Your data will be highly confidential and by completing this survey, you agree to the use of your data in our research. Sex: *Male  *Female Clinical year: *Third  *Fourth *Fifth 12. What do you think are the barriers that prevent the student from reporting any intimidation? You can choose more than one option (A) Fear of shame (B) Fear of society (C) Fear of losing grades by the doctors (D) No response from the official (E) Others like: …………… 13. Do you want to add something about staff intimidation and harassment?

	Always	Frequently	Sometimes	Rare	Never
1. How often do you hear about staff intimidation?					
2 How often do you think occurs at Taibah University?					
3. How often has any doctor shouted or yelled at you?					
4. How often has any doctor belittled or humiliated you?					
5. How often has any doctor made negative or disparaging remarks about your future profession or career in science?					
6. How often has any doctor threatened to harm you?					
7. How often has any doctor slapped, pushed, kicked, or hit you?					
8. How often has any doctor assigned you tasks, work, or rotation responsibilities as a punishment rather than for educational purposes?					
9. How often has any doctor threatened to fail you unfairly or give you an unjustifiably bad grade?					
10. How often has any doctor subjected you to sexual harassment?					
11. If you encounter any future intimidation, how often would you report that incident?					
